# AIEgen-Based Bionic Nanozymes for the Interventional Photodynamic Therapy-Based Treatment of Orthotopic Colon Cancer

**DOI:** 10.1021/acsami.2c04210

**Published:** 2022-05-11

**Authors:** Yanhong Duo, Meng Suo, Daoming Zhu, Zihuang Li, Zheng Zheng, Ben Zhong Tang

**Affiliations:** † Department of Radiation Oncology, The Second Clinical Medical College of Jinan University, 1st Affiliated Hospital of Southern University of Science and Technology, Shenzhen People’s Hospital, Shenzhen 518020, China; ‡ School of Science and Engineering, Shenzhen Institute of Aggregate Science and Technology, The Chinese University of Hong Kong, Shenzhen, Guangdong 518172, China; § School of Chemistry and Chemical Engineering, Hefei University of Technology, Hefei 230009, China; ∥ AnHui Province Key Laboratory of Chemistry for Inorganic/Organic Hybrid Functionalized Materials, Anhui University, Hefei 230601, China; ⊥ Department of Electronic Science and Technology, School of Physics and Technology, Wuhan University, Wuhan 430072, China; # Department of Microbiology, Tumor and Cell Biology (MTC), Karolinska Institutet, Stockholm 17177, Sweden; ∇ Department of Sports Medicine and Rehabilitation, Shenzhen Hospital Peking University, Shenzhen 518036, China

**Keywords:** aggregation-induced emission, interventional photodynamic therapy, platelet-mimicking MnO_2_ nanozyme, tumor targeting, orthotopic colon cancer

## Abstract

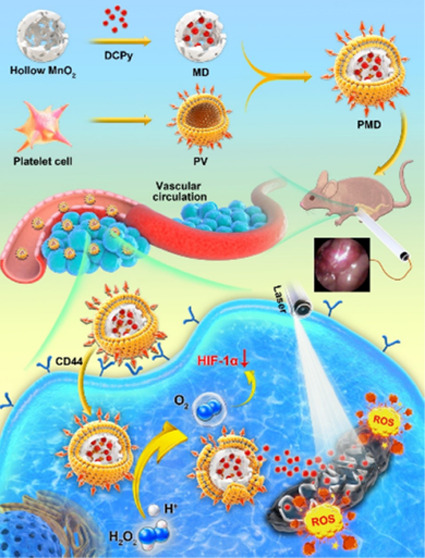

Relative to traditional photosensitizer (PS) agents, those that exhibit aggregation-induced emission (AIE) properties offer key advantages in the context of photodynamic therapy (PDT). At present, PDT efficacy is markedly constrained by the hypoxic microenvironment within tumors and the limited depth to which lasers can penetrate in a therapeutic context. Herein, we developed platelet-mimicking MnO_2_ nanozyme/AIEgen composites (PMD) for use in the interventional PDT treatment of hypoxic tumors. The resultant biomimetic nanoparticles (NPs) exhibited excellent stability and were able to efficiently target tumors. Moreover, they were able to generate O_2_ within the tumor microenvironment owing to their catalase-like activity. Notably, through an interventional approach in which an optical fiber was introduced into the abdominal cavity of mice harboring orthotopic colon tumors, good PDT efficacy was achieved. We thus propose that a novel strategy consisting of a combination of an AIEgen-based bionic nanozyme and a biomimetic cell membrane coating represents an ideal therapeutic platform for targeted antitumor PDT. This study is the first to have combined interventional therapy and AIEgen-based PDT, thereby overcoming the limited light penetration that typically constrains the therapeutic efficacy of this technique, highlighting a promising new AIEgen-based PDT treatment strategy.

## Introduction

Photodynamic therapy (PDT) has emerged as an antitumor therapeutic strategy that enables the targeted treatment of tumors by administering photosensitizer (PS) compounds that generate reactive oxygen species (ROS) following exposure to a particular wavelength of light, thereby inducing significant damage within tumors or other target tissues.^1^ Recent efforts to improve the clinical utility of PDT have focused on developing PS agents exhibiting aggregation-induced emission (AIE) properties.^2−4^ Fluorophores exhibiting AIE characteristics are only minimally emissive when present as isolated molecules, whereas their emissivity rises substantially when they form aggregates.^5,6^ As they are highly biocompatible, photostable, and enable high-contrast imaging, AIE luminogens (AIEgens) have been effectively leveraged for fluorescence-based bioimaging analyses.^7^ As AIEgens can additionally facilitate efficient ROS generation upon exposure to an appropriate wavelength of light, they are also well-suited to use in PDT applications.^7,8^ These AIEgens are more resistant to photobleaching than the traditional PS agents, in addition to being better able to produce a robust ROS response, making them ideal for use in this therapeutic context.

While AIEgens are well-suited to use in PDT-focused applications, this therapeutic modality is nonetheless limited by the inability of light to effectively penetrate deep within tissues. As many of the most severe tumor types are located deep within the abdomen, such as colon, pancreatic, and liver tumors, these malignancies are generally resistant to PDT. AIEgen-based PDT is thus currently restricted to use in the treatment of superficial tumors, greatly restricting its clinical utility. To overcome this limitation, we herein developed an interventional therapeutic strategy whereby deep tumors can be directly subjected to interventional irradiation, overcoming many of the clinical challenges associated with accessing and treating these malignancies.

In addition to being difficult to access in many cases, the tumor microenvironment (TME) is also profoundly hypoxic, which can suppress the efficacy of PDT strategies that are oxygen-dependent.^3^ This hypoxic TME develops due to the irregular cell growth and angiogenic activity that occur within tumors^9^ but can be further exacerbated when PDT induces the collapse of the local microvasculature. This can further hamper the clinical value of PDT owing to the tendency of this approach to rapidly exhaust local oxygen supplies while preventing their efficient restoration.^10,11^ Moreover, the molecular mechanisms whereby AIEgens mediate PDT are incompletely understood and warrant further research. As such, there is a clear need for the development of a new AIEgen-based multifunctional nanoplatform that can efficiently mediate PDT while enabling further study of the mechanistic underpinning of this therapeutic modality.

Recently, a growing body of research has focused on leveraging natural enzymes in therapeutic contexts. In the context of PDT, the oxygen-generating activity of enzymes such as catalase (CAT) has been studied as a potential approach to improving O_2_ availability within the TME.^12,13^ Nanozymes leverage stable, inexpensive, tunable nanoparticles (NPs) to mimic the activities of specific enzymes in diverse therapeutic contexts.^1,14^ MnO_2_ nanozyme-based NPs, for example, have been shown to be capable of decomposing hydrogen peroxide (H_2_O_2_) within the acidic TME, thereby functioning in a manner analogous to CAT and overcoming local intratumoral hypoxia by producing water and oxygen.^15^ Hollow multifunctional MnO_2_ nanozyme NPs have also shown promise as tools for mediating the targeted delivery of drugs into tumors, overcoming local hypoxia and thereby augmenting PDT efficacy.^10,12^ Over time, these nanozymes will accumulate within the kidneys wherein they will be degraded and cleared without inducing significant tissue toxicity. As the encapsulation efficiency (EE) of hollow MnO_2_ NPs is very high, they are well-suited to the intratumoral delivery of particular drugs.^16^ AIEgen-based MnO_2_ NPs may therefore be well-suited to overcoming the limited solubility of traditional PS agents while simultaneously overcoming the hypoxic environment within tumors, thus synergistically enhancing PDT efficacy. Despite their promising properties, MnO_2_-based NPs do not have the ability to intrinsically target tumors *in vivo*, necessitating their further surface modification. Circulating platelets (PLTs) play critical roles in regulating pathogen responses and vascular damage repair^17^ and can also interact with circulating tumor cells (CTCs) that they hold great promise for targeting or interfering with tumor metastatic progression.^18,19^ Notably, these PLT membranes can be readily collected and used for NP coating, thereby enabling the generation of biomimetic NPs that are better able to specifically traffic to sites of vascular damage and tumors while avoiding NP-induced complement activation and evading macrophage-mediated phagocytosis of these particles.^20^ Owing to the promising properties of such membrane-coated particles, we herein employed novel PLT membrane-coated bionic nanozymes in an effort to improve the efficacy of PDT when employed to treat hypoxic tumors.

**Scheme 1:**
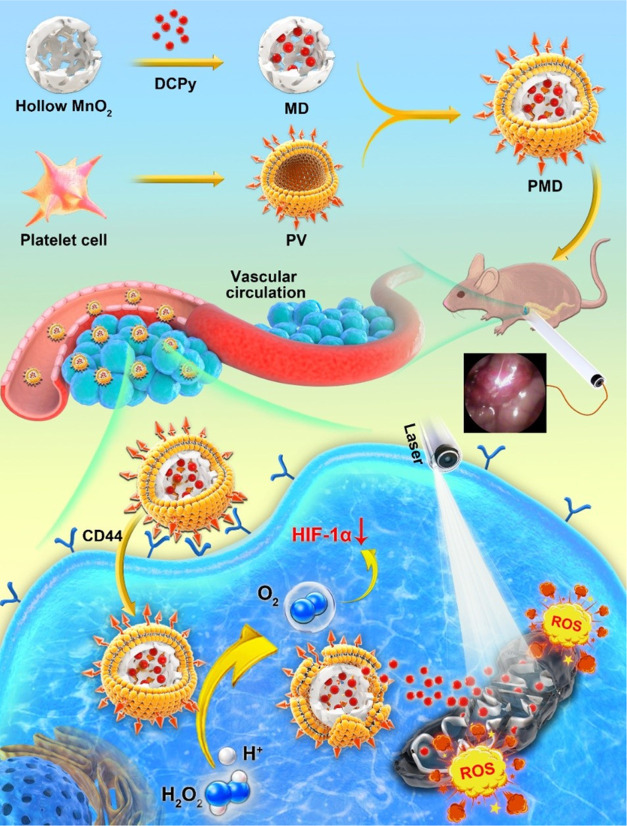
Schematic Overview of the Use of AIEgen-Based Bionic Nanozymes for the Interventional Photodynamic Therapy Against Orthotopic Colon Cancer

Here, we describe the development of AIEgen-based bionic nanozymes that were successfully employed for the interventional PDT treatment of hypoxic tumors (Scheme 1). For this approach, 2,6-dichloropyridine (DCPy) was utilized as an AIE PS given that it can efficiently mediate PDT, as discussed in prior reports.^21^ By coupling DCPy to hollow MnO_2_ NPs, we successfully generated MnO_2_/DCPy (MD) NPs that were then coated with PLT-derived vesicles (PVs) collected from samples of murine blood, thus producing PLT-coated bionic nanozyme (PMD) particles. These PMD preparations were able to efficiently target tumor tissues and mediated good PDT efficacy when employed together with an interventional treatment approach in a murine orthotopic colon cancer model system. These PMD particles exhibited superior efficacy to red blood cell (RBC) membrane-coated MD (RMD) NPs owing to their more efficient tumor-targeting and antihypoxic activity. The bionic nanozymes employed in this approach have the potential to overcome many of the hurdles to effective AIEgen deployment when performing PDT, thereby facilitating superior tumor growth inhibition. This study is the first, to our knowledge, to have combined interventional therapy and AIEgen-based PDT, overcoming the limited light penetration associated with traditional PDT, thereby highlighting a promising new therapeutic modality worthy of further study.

## Results and Discussion

**Figure 1:**
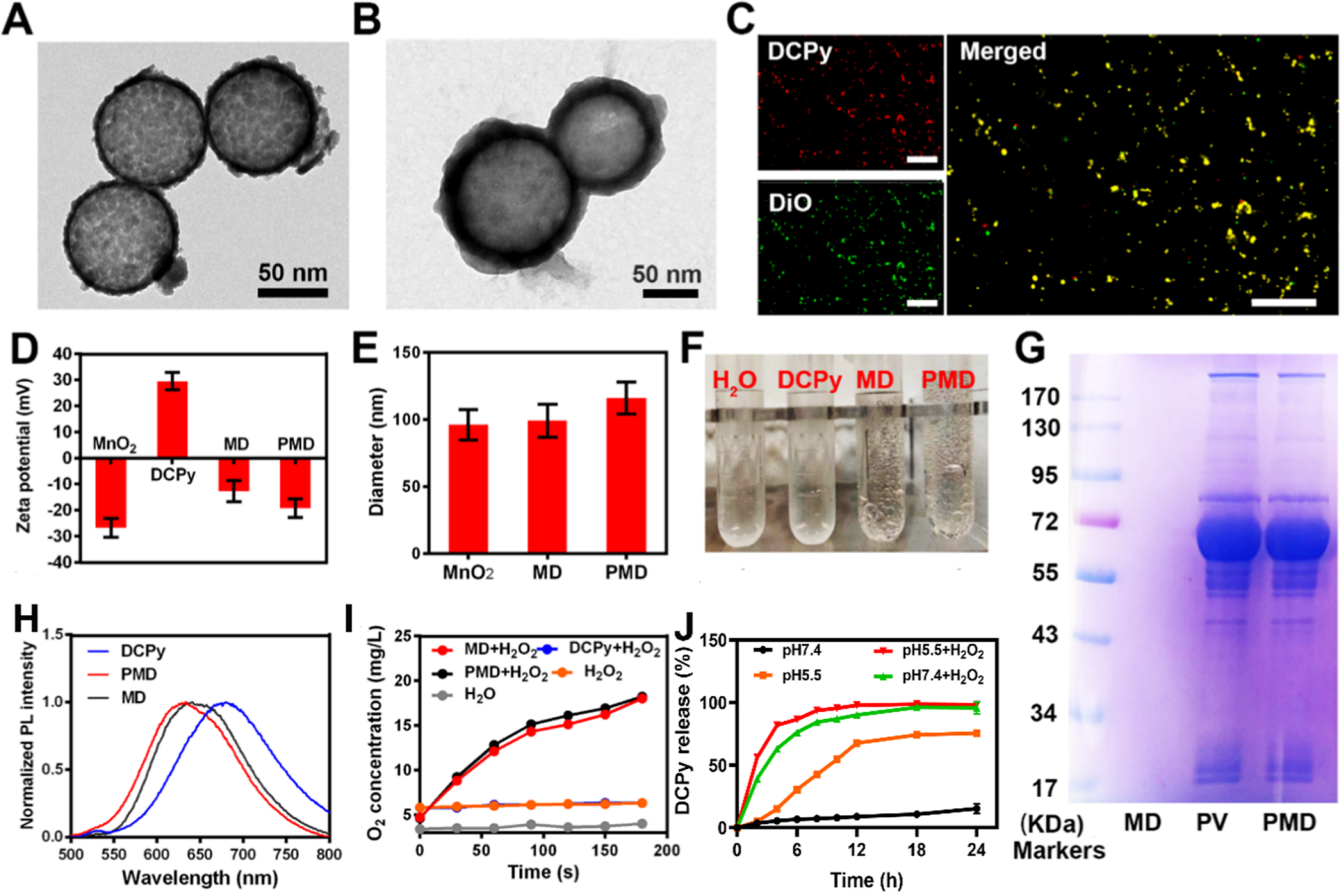
Representative TEM images of (A) MnO_2_ and (B) PMD preparations. (C) Confocal microscopy was used to assess the colocalization of DiO (green) and DCPy (red) in PMD samples. Scale bar: 5 μm. (D) MnO_2_, DCPy, MD, and PMD ζ-potential values were measured. (E) MnO_2_, DCPy, MD, and PMD particle hydrodynamic diameter values were measured via DLS. (F) Ability of the indicated samples to catalyze H_2_O_2_ decomposition was assessed. (G) Proteins present in MD, PMD, and PV samples were analyzed via SDS-PAGE. (H) Normalized PL spectra for PMD (in PBS), MD (in PBS), and DCPy aggregates (in 90% water, 10% DMSO). (I) Ability of DCPy, MD, and PMD samples to generate oxygen when combined with H_2_O_2_ was quantified. (J) Cumulative release of DCPy from PMD was assessed under the indicated conditions.

Initially, DCPy was synthesized according to the reported procedure.^21^^1^H NMR spectrum (Figure S1),^13^C NMR (Figure S2) spectrum, and high-resolution mass spectrometry (HRMS) (Figure S3) were used for the characterization of DCPy. Nanozyme/AIEgen composites (MD) were then synthesized via mechanical mixing,^10^ and the prepared MD and sSiO_2_ NP samples were characterized via transmission electron microscopy (TEM), which revealed them to exhibit an average diameter of ∼100 nm. PMDs were subsequently prepared and found to possess an outer shell that was 10 nm-thick, consistent with membrane encapsulation having been performed successfully (Figures 1A,B and S4). MD particles exhibited a DCPy EE of 71.1 ± 3.9%, with this rate being higher than that observed for standard polymer NPs.^2,6^ We additionally used UV–vis spectrometry, dynamic light scattering (DLS), sodium dodecyl-sulfate polyacrylamide gel electrophoresis (SDS-PAGE), and fluorescence localization assays to confirm that PMD and DCPy had been successfully coupled. In these assays, PMD particles were found to exhibit characteristic red fluorescence consistent with the properties of DCPy and were effectively stained by 3,3′-dioctadecyloxacarbocyanine perchlorate (DiO), which is a green fluorescent membrane dye (Figure 1C). While MnO_2_ NPs (average diameter: 96.0 nm; PDI: 0.106) and MD (average diameter: 99.0 nm; PDI: 0.122) were similarly sized (Figure 1E), PMD particles were larger (average diameter: 116.0 nm; PDI: 0.140), further supporting their successful encapsulation (Figure 1E). PMD particles were highly stable and did not exhibit any changes in size when cultured in phosphate-buffered saline (PBS) or fetal bovine serum (FBS) for 3 days at 4 °C (Figure S5). The ζ-potential values of MD and PMD NPs exhibited similar trends (Figure 1D). An X-ray diffractometer (XRD) and X-ray photoelectron spectroscopy (XPS) additionally confirmed the successful synthesis of a pure NP solution (Figures S6 and S7). The CAT-like nanozyme properties of the prepared MnO_2_ samples were further confirmed through analyses indicating that while DCPy was unable to decompose H_2_O_2_, both MD and PMD preparations were able to efficiently generate O_2_ over time in this assay context (Figure 1F,I). The PV coating of these MD particles thus had no adverse impact on their nanoenzymatic activity. The ζ-potential of MD NPs was reduced relative to that of pure MnO_2_ NPs, likely owing to positively charged DCPy adsorption to NP surfaces. PMD samples exhibited high levels of PLT-derived proteins (Figure 1G). P-selectin is a protein that is expressed at high levels on platelets and binds to CD44 with very high affinity. As such, we evaluated P-selectin levels in these PMD preparations and found its expression to be comparable to that observed in PV samples (Figure S8). This result is promising, as the expression of CD44 by tumor cells can thus facilitate the targeting of P-selectin-coated PMD particles to tumor sites, thereby promoting superior PDT efficacy.^18^ Analyses of the photoluminescence (PL) spectra of prepared PMD samples (in PBS) and free DCPy (aggregate in aqueous 1% dimethyl sulfoxide, DMSO) revealed respective peaks at 633 and 698 nm (Figure 1H); this blue-shifted emission of DCPy in PMD particles might be attributed to the less polar environment inside the hollow MnO_2_ NPs. When exposed to acidic conditions in the presence of H_2_O_2_ and H^+^, DCPy was readily released from PMD NPs (Figure 1J), while its release was largely absent in neutral PBS. Similarly, TEM analyses demonstrated the efficient degradation of PMD particles when suspended in an acidic buffer (pH 5.5) supplemented with 100 μM H_2_O_2_ (Figure S9).

**Figure 2:**
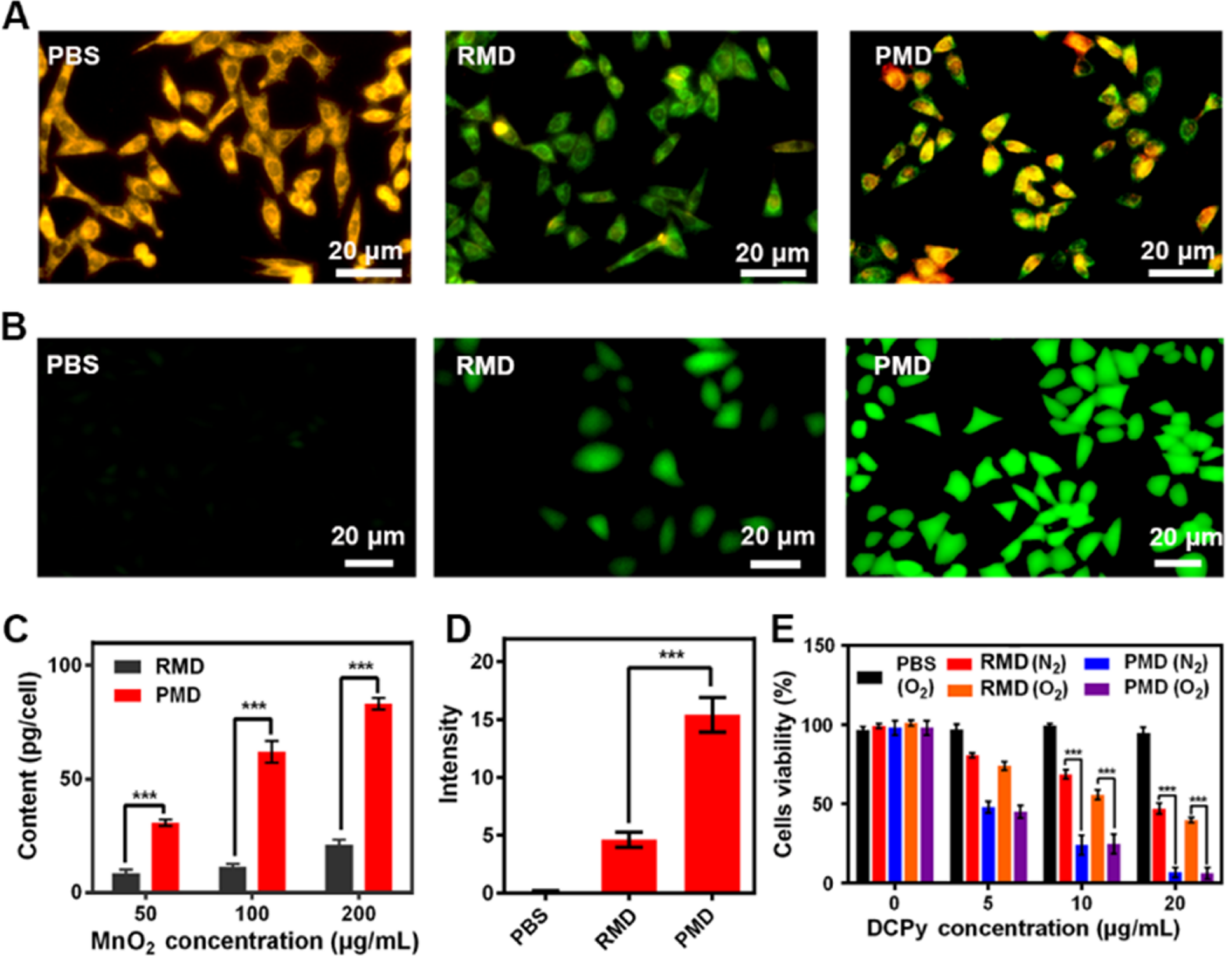
(A) Representative confocal laser scanning microscopy (CLSM) images of CT26 cells after treatment for 30 min with DCPy, RMD, or PMD. Green: Mito-tracker green, red: DCPy. (B) 2’-7’-Dichlorofluorescin diacetate (DCFH-DA) was used to stain CT26 cells that had been treated as indicated under hypoxic conditions. (C) Uptake of the indicated concentrations of NPs by CT26 cells was evaluated. (D) DCFH-DA fluorescence intensity quantification. (E) CT26 cell viability was assessed via the MTT assay after treatment with PBS, RMD, or PMD under normoxic or hypoxic conditions (***P < 0.001, Student’s *t*-test).

As detailed above, the coating of nanoparticles with the PLT-derived membrane has the potential to significantly improve their therapeutic utility by enhancing their ability to adhere to tumor tissues while reducing their uptake by macrophages.^18^ To confirm that this holds true for our PMD preparations, we incubated MD and PMD samples with murine macrophages and then quantified their internalization of MnO_2_ via an inductively coupled plasma optical emission spectrometer (ICP-OES). These analyses revealed that dose-dependent MnO_2_ uptake was significantly more robust for macrophages incubated with MD NPs relative to those incubated with PMD NPs (Figure S10), thus confirming the ability of these biomimetic particles to better evade phagocytic internalization. We additionally prepared RBC membrane-coated MD (RMD) particles to compare their tumor-targeting activity to that of PMD particles (Figure S11). Fluorescence microscopy revealed that PMD NPs were able to target CT26 cells *in vitro* in assays in which these RMD-, PMD-, or DCPy-treated cells were stained using the mitochondrial probe Mito-tracker Green FM (Figure 2A). Within 30 min of treatment, PMD attachment to tumor cells was evident, whereas DCPy staining within mitochondria was also observed after 30 min, consistent with prior evidence that DCPy can stain mitochondria within live cells.^21^ These results were thus consistent with the ability of PMD particles to effectively target tumor cells and to deliver their payload therein. The internalization of PMD and RMD particles was further assessed by incubating these particles with CT26 cells for 30 min and then measuring intracellular Mn levels as above. This experiment revealed significantly increased Mn^2+^ levels within PMD-treated cells relative to RMD-treated cells (Figure 2C), supporting the ability of PLT-derived membranes to enhance the tumor-targeting activity of these NPs.

Effective nanoscale therapeutic platforms need to induce minimal toxicity and to exhibit a high degree of biocompatibility.^22,23^ As prior research suggests that MnO_2_ is cytotoxic, we evaluated its effects on a range of cancer cells and observed marked cytotoxic cell death at an MnO_2_ dose of 20 μg/mL (Figure S12). However, these NPs did not induce any substantial cytotoxicity when incubated with other cell lines, consistent with their satisfactory biocompatibility (Figure S13). PMD particles failed to induce hemolysis of rabbit blood at any tested concentration levels (Figure S14). Intratumoral ROS staining was further performed using the fluorescent DCFH-DA probe (Figure 2B,D), revealing that weak ROS generation (green fluorescence) was evident in hypoxic tumor cells following DCPy treatment, whereas PMD treatment markedly enhanced such ROS production. Together, these results suggested that PMD can induce the robust generation of ROS within tumor cells under hypoxic conditions. We then further compared the relative efficacy of PDT performed using DCPy and PMD preparations in hypoxic and normoxic contexts (Figure 2E). When the laser-induced phototoxic death of cells in these different treatment groups was assessed, RMD treatment at a 20 μg/mL DCPy dose resulted in 47% cell viability, whereas just 6% of cells remained viable following laser irradiation when treated with PMD particles at an equivalent DCPy dose. Under normoxic conditions, both PMD and RMD particles effectively inhibited tumor growth. PMD particles were thus better suited to resisting the adverse effects of hypoxia when facilitating PDT, likely owing to the decomposition of H_2_O_2_ facilitated by MnO_2_ (Figure S15) and the associated increase in oxygen levels within cells.^10^ Relative to RMD particles, PMD particles exhibited superior therapeutic efficacy consistent with the ability of PMD to better target tumor cells and to thereby more readily induce tumor cell death.

**Figure 3:**
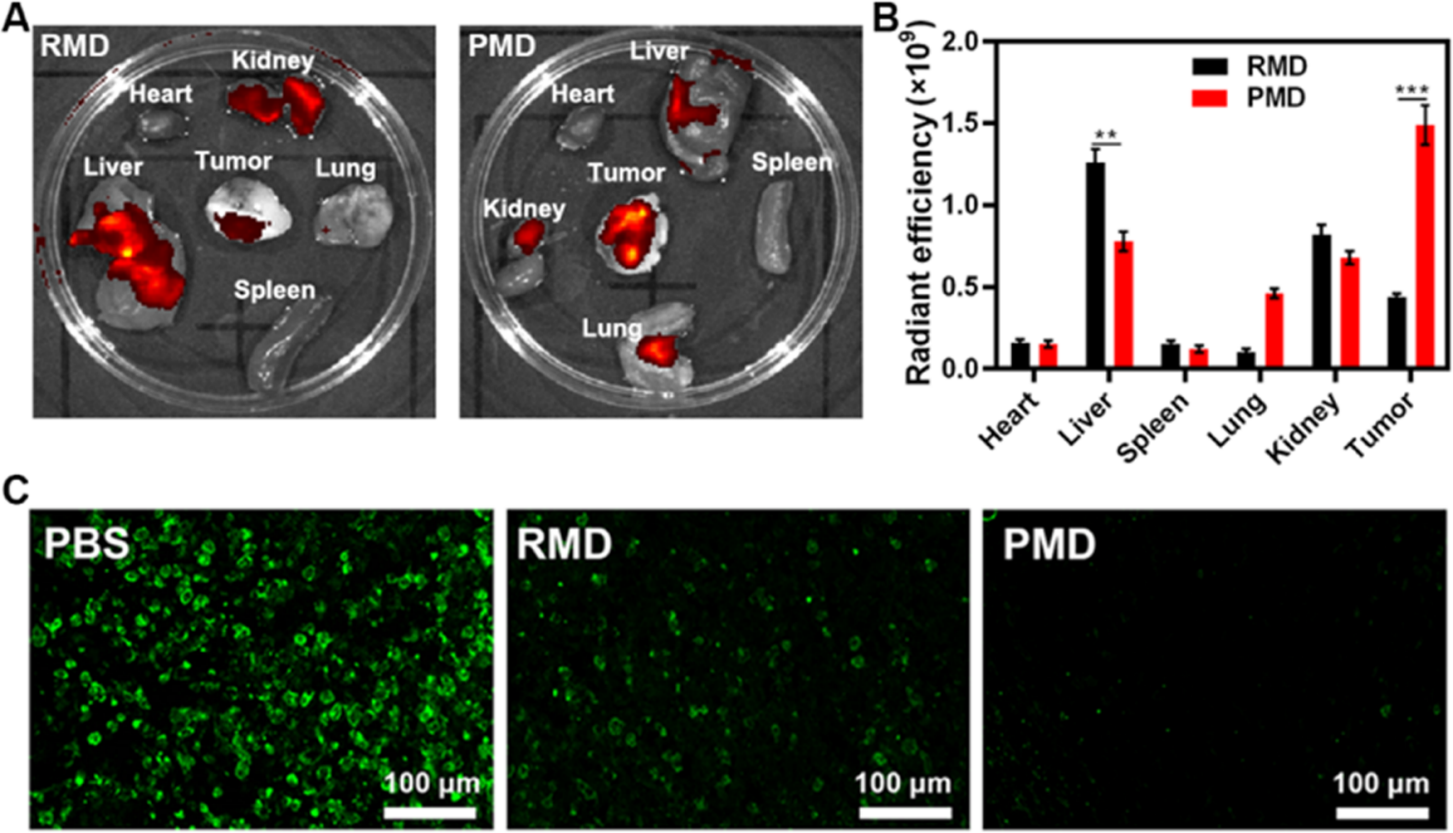
(A) *Ex vivo* images and (B) radiant efficiency in tumors and harvested organs from orthotopic CT26 tumor-bearing mice 6 h following PMD or RMD injection. (C) Fluorescent staining for intratumoral hypoxia (green: HIF-1α). (**P < 0.01, ***P < 0.001; Student’s *t*-test).

Next, CT26 tumor-bearing mice were intravenously injected with RMD or PMD preparations containing equivalent DCPy doses to assess the *in vivo* biodistribution profiles of these particles. Higher levels of PMD accumulation were evident within tumors as compared to those observed for RMD (Figure 3A,B), confirming the superior tumor-targeting activity of PMD particles. Note that tumors were analyzed *ex vivo* owing to the high levels of background fluorescence produced by the intestines in this assay system. Given the ability of intratumoral hypoxia to limit PDT efficacy,^24^ the TME hypoxia was next examined in these tumor-bearing mice (Figures 3C and S16). At 6 h post-treatment with PBS, RMD, or PMD (10 mg/kg equivalent MnO_2_ dose), mice were euthanized and tumors were collected to stain for hypoxia-inducible factor (HIF-1α) levels within the TME. Robust HIF-1α staining was observed in control animals (≈74% HIF-1α positive), consistent with profound intratumoral hypoxia. This staining was decreased in RMD-treated mice (≈37% HIF-1α positive) and was reduced even further in animals treated with PMD (≈6% HIF-1α positive). The observed differences between the RMD and PMD treatments were likely attributable to the improved tumor targeting of PMD particles.

**Figure 4:**
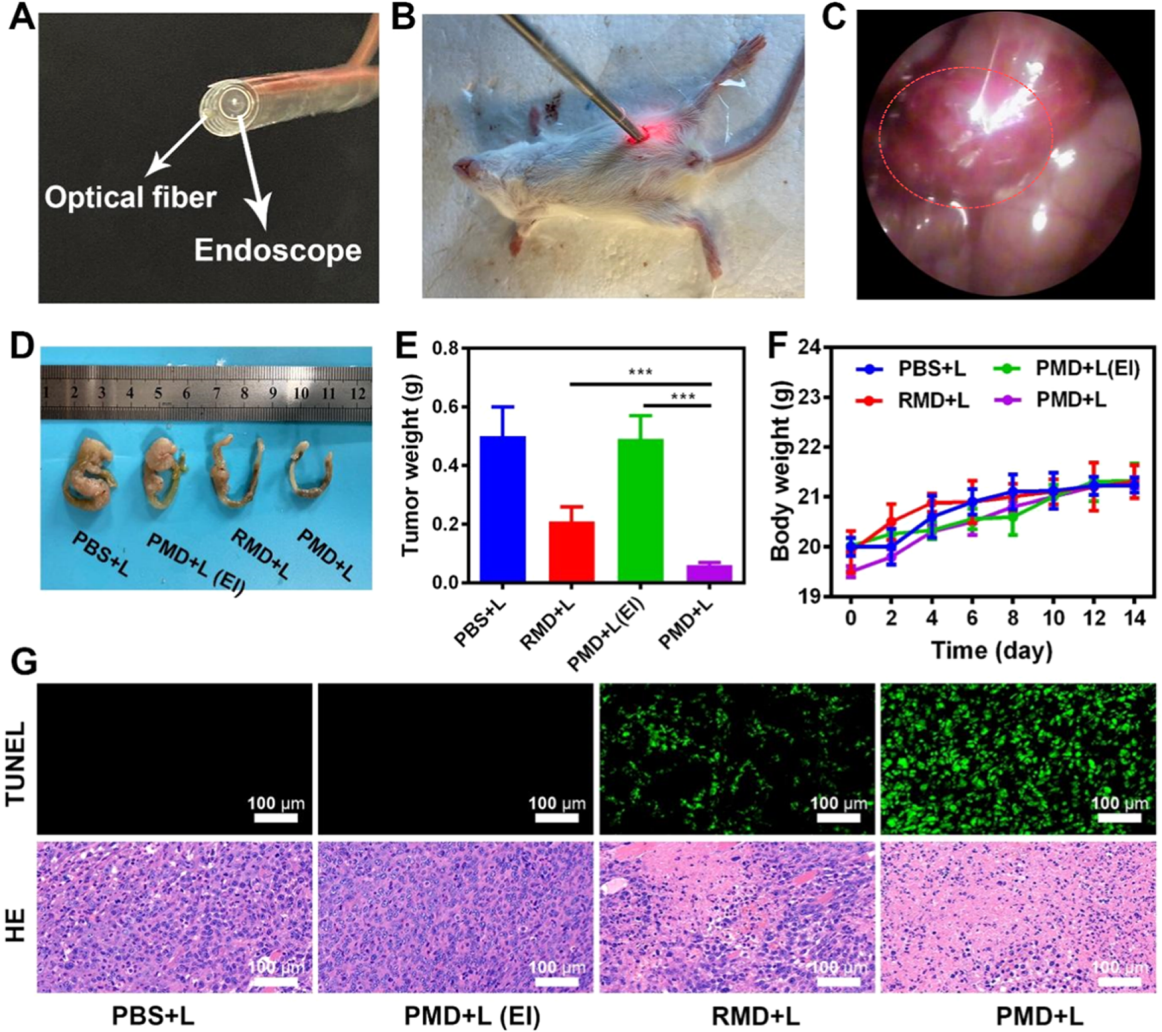
(A) Immunofluorescent staining of tumors in the indicated treatment groups was conducted. Scale bar: 100 μm. (B) Immunofluorescent HIF-1α staining was quantified, with differences among groups being assessed via Student’s *t*-test. (C) Tumor blood oxygen saturation prior to and following the indicated intravenous treatments. (D) Tumor growth, (E) final tumor weight, and (F) body weight were monitored in different treatment groups. (G) Tumor sections in the indicated groups were subjected to H&E and DCFH-DA staining. Scale bar: 100 μm (*P < 0.05, **P < 0.01, ***P < 0.001; Student’s *t*-test).

To evaluate the ability of our PMD preparations to facilitate PDT *in vivo*, we next developed an interventional treatment strategy for colon tumors located deep within the abdominal cavity. This approach utilized an interventional device consisting of an optical fiber and an endoscope joined by a medical tape (Figure 4A).^25^ During treatment, the abdominal cavity of a given mouse was punctured with an appropriate needle that was then extracted, with the interventional device then being inserted into the abdomen through the resultant hole. The endoscope was then used to locate the tumor within the abdominal cavity, and a laser was used to initiate PDT treatment via the optical fiber (Figure 4B). Orthotopic tumors were clearly visible within the abdomen under endoscopic visualization (Figure 4C, red dotted line). Note that the laser appears red in some images (Figure 4B), likely owing to light shining on the local vasculature. When tumors were close in size to the diameter of the intestines, interventional treatment was initiated every 4 days in these tumor-bearing mice. No animals died during treatment, consistent with the safety of this therapeutic modality. While tumors in untreated mice grew rapidly, combination PMD + L treatment was associated with significantly increased antitumor activity that was superior to that observed for RMD + L treatment (Figure 4D). PLT-derived membranes are thus well-suited to achieving better therapeutic outcomes when used to coat NPs owing to the ability of the resultant particles to traffic to tumors and to decrease intratumoral hypoxia. Notably, negligible therapeutic efficacy was observed in the PMD + L (EI) treatment group in which external laser irradiation was performed, consistent with the inability of the low-power laser utilized in these experiments to penetrate the abdominal cavity to facilitate PDT. In contrast, interventional therapy achieved robust efficacy, underscoring the promise of this treatment approach. No significant changes in murine body weight were observed over the study period, consistent with the safety of the administered NPs (Figure 4F). Moreover, significant tumor damage was evident following both hematoxylin and eosin (H&E) and TUNEL staining in the PMD + L treatment group (Figure 4G). To further confirm the safety of this therapeutic modality, major organs were collected from treated mice, and overall H&E-stained tissue morphology was assessed. No evidence of pathological lesions was observed in kidney, liver, heart, lung, or spleen samples from PMD-treated mice as of day 14 post-treatment (Figure S17), further confirming that these NPs do not induce significant *in vivo* toxicity. Overall, these results suggest that the developed biomimetic nanozyme/AIEgen platform is an effective approach to facilitating *in vivo* PDT.

## Conclusions

In conclusion, we herein developed a platelet-mimicking nanozyme/AIEgen composite capable of facilitating the interventional photodynamic treatment of hypoxic tumors. This biomimetic nanozyme platform was able to overcome many of the challenges previously associated with the use of AIE photosensitizer agents when conducting PDT, including the limited depth of penetration for lasers utilized in this therapeutic context and the high levels of hypoxia within most solid tumors. This study is the first to report the use of a combination of interventional therapy and AIEgen-based PDT, and our promising results highlight the clinical potential of this and other novel AIEgen-based therapeutic strategies. These biomimetic nanozyme/AIEgen systems thus hold great promise for the clinical treatment of a broad array of cancer types. In future studies, we will combine interventional therapy approaches and targeted treatment strategies to more effectively treat other hypoxic abdominal tumor types.

## Experimental Section

### Materials

Tetraethyl orthosilicate (TEOS) and calcein were obtained from Aladdin Reagent (Shanghai, China). Potassium permanganate (KMnO_4_) and sodium carbonate (Na_2_CO_3_) were from Sinopharm Chemical Reagent Co., Ltd. (China). DCPy was synthesized according to the reported procedure.^21^ PBS was from Thermo-Fisher (MA). Mito-Tracker Green, 3,3′-dioctadecyloxacarbocyanine perchlorate (DiO), and a Reactive Oxygen Species Assay Kit were from Beyotime Company (China). Deionized (DI) water prepared with a purification system (Direct-Q3, Millipore) was used when formulating aqueous solutions, while all other solvents were from Sinopharm Chemical Reagent (China) and Aladdin Reagent (China).

### Instrumentation

A white light laser consisting of three different wavelengths of light and an optical fiber 1 mm in diameter were purchased from Ningbo Yuanming Laser Technology Co., Ltd. (China). A medical endoscope was obtained from Le Weishi Technology Co., Ltd. (China). Medical tape was used to link the endoscope to the optical fiber. An 18-gauge PTC needle (Zhuhai Hokai Biomedical Electronics Co., Ltd., China) was used to penetrate the abdominal cavity when performing interventional treatment, after which the optical fiber and endoscope were inserted into this cavity for therapeutic use.

### Cell Culture

CT26 mouse colon cancer cells, RAW 264.7 murine monocytic macrophages, MCF-7 human breast cancer cells, and U937 human macrophages were purchased from the Cell Bank of the Chinese Academy of Sciences. All cells were cultured in RPMI-1640 containing 10% FBS in a humidified 37 °C incubator. For normoxic culture (pO_2_: 21%), cells were cultured in the presence of 5% CO_2_ and 95% air, whereas for hypoxic culture (pO_2_: 2%), cells were cultured in a hypoxic incubator (Moriguchi, Japan) in 2% O_2_, 5% CO_2_, and 93% N_2_.

### Orthotopic Colon Tumor Model Establishment

The Institutional Ethics Committee for Animal Experimentation approved all murine experimental protocols, which were consistent with the guidelines established by Shenzhen People’s Hospital (Second Clinical Medical College of Jinan University). To establish orthotopic tumor models, Balb/c mice were intraperitoneally injected with 1% pentobarbital to establish anesthetization, after which they were fixed in the supine position on sterile gauze. The hair overlying the operative site was removed via shaving, after which iodophor and 95% alcohol were sequentially used to sterilize the skin. A longitudinal incision was then made at the lower margin of the abdominal line, with the skin and peritoneal layers then being incised in a layer-by-layer manner to expose the bladder, which was removed with forceps along with the small intestine. A segment of the large intestine deep within the abdomen was then located, and a handheld syringe was used to inject 25 μL of CT26 cells (1 × 10^8^ cells/mL) into the colon wall in a direction parallel to its surface, with the colon being held using ophthalmic tweezers. The injection was targeted to penetrate the serosal layer, thereby injecting cells into the colon submembrane. After injection, the needle was allowed to remain in place briefly, after which it was carefully withdrawn, and sterile gauze was applied to the injection site for 10 s to prevent the liquid therein from leaking out. The abdominal cavity was then closed.

### Hollow MnO_2_ NP Synthesis

Solid silica NPs (sSiO_2_) were prepared as reported previously.^10^ An aqueous KMnO_4_ (300 mg) solution was then combined with the suspended sSiO_2_ NPs (40 mg) in a dropwise manner with ultrasonication. Following a 6 h incubation, samples were centrifuged at 14,800 rpm, and precipitates were collected via centrifugation. The resultant mesoporous MnO_2_-coated sSiO_2_ was dissolved in 2 M aqueous Na_2_CO_3_ for 12 h at 60 °C. The resultant hollow mesoporous MnO_2_ NPs were then collected via centrifugation and washed repeatedly with water.

### DCPy-Loaded MnO_2_ (MD), Red Blood Cell (RBC) Membrane-Coated MD (RMD), and Platelet Membrane-Coated MD (PMD) Nanoparticle Preparation

Platelet (PLT)- and red blood cell (RBC)-derived vesicles (PVs and RVs) were prepared as reported previously.^18,23^ MnO_2_ NPs (10 mg) were loaded with DCPy by dispersing them in a 6 mL mixture of DMSO saturated with DCPy (5 mg/mL), followed by stirring for 24 h while being protected from light. NPs were then collected via centrifugation (25 min at 12,000 rpm) and resuspended in ultrapure water. DCPy EE was calculated by measuring unloaded DCPy concentrations via UV–vis spectrophotometry, with the resultant EE being determined as follows: EE = initial drug weight – remaining drug weight/(initial drug weight) × 100%.^23^ The PV and RV coatings of these NPs were achieved by mixing an MD solution (5 mL, 1 mg/mL) with appropriate vesicles (5 mL, 1 mg/mL) and then extruding the resultant solution 11 times through a 200 nm polycarbonate porous membrane using an Avanti mini extruder.^19^ The resultant PMD or RMD preparations were then collected via centrifugation.

### PMD Nanoparticle Characterization

MD, RMD, and PMD nanoparticle morphologies were assessed via transmission electron microscopy (TEM; JEOL-2100). The hydrodynamic diameters and ζ-potential values of these particles were determined via dynamic light scattering (Nano-ZS ZEN3600). A Fluorolog-3 fluorescence spectrophotometer (Horiba Jobin Yvon Inc., France) was used for measurements of the photoluminescence (PL) spectra for these particles.

### Drug Release and Degradation Analyses

DCPy release was assessed by dialyzing a PMD solution against PBS at a pH of 5.5 or 7.4 in the presence or absence of H_2_O_2_, with DCPy release over time being measured via UV–vis spectrophotometry.

### Oxygen Generation Analysis

PBS, DCPy, MD, and PMD samples (DCPy concentration: 10 μM) were resuspended in 3% H_2_O_2_ or ultrapure water (8 mL), after which the oxygen concentrations in these solutions were monitored in real time using a DOG-3082-dissolved oxygen meter.

### Western Blotting

A protein extraction kit (Dingguo, China) was used to isolate cellular proteins, which were separated via SDS-PAGE. The resultant gels were stained with Coomassie Blue, and proteins were then transferred to PVDF membranes (Bio-Rad). After blocking with 5% nonfat milk for 1 h, blots were incubated overnight with anti-P-selectin (Proteintech) at 4 °C, followed by incubation for 1 h at room temperature with an appropriate secondary antibody.

### SDS-PAGE Protein Analysis

Proteins were analyzed via SDS-PAGE. Briefly, MD, PC, and PMD samples were suspended in the SDS sample buffer at concentrations determined based on a BCA assay kit. Samples were then warmed to 95 °C for 5 min, after which 20 μg per sample was separated via 10% SDS-PAGE for 2 h at 120 V, with the resultant gels being stained using Coomassie Blue for 2 h, washed overnight, and imaged.

### *In Vitro* Immune Evasion Analyses

After culturing for 12 h in 12-well plates, RAW 264.7 cells were treated with a range of MD or PMD concentrations (50, 100, or 200 μg/mL MnO_2_) for 2 h at 37 °C under normoxic conditions. Cells were then rinsed thrice using PBS, after which NP uptake was quantified by ICP-OES. Mn uptake by these cells was measured by adding 0.5 mL of 1% Tween-80 per well to lyse the cells, with lysates then being treated using concentrated nitric acid and 30% H_2_O_2_ (1:2), with the resultant solution then being allowed to rest for 12 h at room temperature in an airing chamber, followed by incubation for 6 h in an oil bath at 80 °C to remove acids. The remaining Mn levels in these samples were then quantified via an inductively coupled plasma optical emission spectrometer (ICP-OES).

### *In Vitro* Tumor-Targeting Analyses

After culture for 12 h in 24-well plates, CT26 cells were treated with a range of RMD or PMD concentrations. After incubation for 30 min at 37 °C under normoxic conditions, cells were washed thrice with PBS, fixed for 30 min at room temperature with paraformaldehyde (PFA), stained using Mito-tracker Green, and imaged via a confocal laser scanning microscope (CLSM; IX81, Olympus, Japan). NP uptake was then assessed via ICP-OES as above.

### PMD Biocompatibility Analysis

PMD biocompatibility was assessed by playing Huh-7, CT26, MCF-7, or RAW 264.7 cells in 96-well plates (5 × 10^3^ cells/well) for 24 h, after which cells were treated with a range of concentrations (0, 5, 10, or 20 μg/mL MnO_2_ and DCPy) for 6 h. Then, MTT reagent (5 mg/mL in PBS) was added to each well, and the cells were incubated for an additional 4 h. Absorbance at 570 nm was then measured via microplate reader (*E*_max_ Precision), with background absorbance being removed via subtraction. Cytotoxicity was measured based on the absorbance (OD) value in the treatment (T) group divided by the OD of the control (C) group (T/C × 100%).

### Hemolysis Assay

The *in vitro* cytotoxicity of PMD preparations was further assessed via a hemolysis assay. Briefly, 5 mL of EDTA-anticoagulated (0.2 mL) rabbit heart blood was obtained, and the RBCs therefrom were collected via centrifugation and washed with PBS (2%). Equal volumes of RBC solution and PMD in PBS (0.5 mL each) were then mixed at different concentrations (5, 10, or 20 μg/mL DCPy). Water and PBS respectively served as positive and negative control treatments. Samples were gently mixed, after which they were incubated for 3 h at room temperature. Absorbance at 570 nm in the supernatant fraction of each sample was then measured with a UV–vis photospectrometer. The hemolysis ratio was determined as follows: hemolysis ratio = (sample absorbance – negative control absorbance)/(positive control absorbance – negative control absorbance) × 100.

### Intracellular ROS Generation Assay

To analyze ROS generation, CT26 cells were treated for 2 h under three different conditions: (1) PBS, (2) RMD, or (3) PMD. In groups 2–3, a DCPy concentration of 10 μM was utilized. All cells had been precultured under hypoxic conditions. The fluorescent DCFH-DA dye (10 μM) was then added to wells, and cells were cultured for an additional 20 min at 37 °C prior to white light irradiation (400–700 nm, 5 min, power density: 0.1 W/cm^2^). ROS levels were detected via confocal laser scanning microscopy (CLSM; IX81, Olympus, Japan), with ImageJ being used to quantify fluorescence intensity values in individual groups.

### Analysis of PMD Phototoxicity

An MTT assay was employed to examine the phototoxicity of the prepared nanoparticles. Briefly, CT26 cells were added to 96-well plates (5 × 10^3^/well) for 24 h, after which the cells were assigned to five different treatment groups: (1) PBS (O_2_); (2) RMD (O_2_); (3) RMD (N_2_); (4) PMD (O_2_); and (5) PMD (N_2_). In groups 2–5, a DCPy concentration of 10 μg/mL was used. Cells were cultured under either normoxic (O_2_) or hypoxic (N_2_) conditions as detailed in the cell culture section above, with hypoxic preconditioning having been achieved by initially incubating cells for 12 h in a hypoxic incubator (1% O_2_, 5% CO_2_, and 94% N_2_). White light (400–700 nm) was used to irradiate cells in appropriate groups for 5 min (power density: 0.1 W/cm^2^). At appropriate time points, MTT reagent (5 mg/mL in PBS) was added, and plates were incubated for an additional 4 h. Absorbance at 570 nm was then measured via a microplate reader (*E*_max_ Precision), with background absorbance being removed via subtraction. Cytotoxicity was measured based on the OD value in the treatment (T) group divided by the OD of the control (C) group (T/C × 100%). This same approach was used to confirm the *in vitro* phototoxicity of PMD using a range of DCPy concentrations (5 or 20 μg/mL).

### *In Vivo* Distribution Analysis

When orthotopic CT26 tumors were up to 7 mm in diameter (twice the intestinal diameter) as measured via endoscopy, tumor-bearing mice (*n* = 3) were intravenously injected with 100 μL of RMD or PMD in PBS (DCPy dose: 5 mg/kg). At 6 h post-injection, mice were euthanized, and tumors and major organs were collected to assess Cy5 distributions and fluorescence intensity values with the IVIS system. Owing to the strong background fluorescence of the intestinal tract in the analyzed range, tumors were isolated for *in vivo* imaging.

### Analysis of *In Vivo* Tumor Hypoxia

When orthotopic CT26 tumors were up to 3 mm in diameter, as measured via endoscopy, tumor-bearing mice (*n* = 3) were intravenously injected with 100 μL of RMD or PMD in PBS (DCPy dose: 5 mg/kg). At 6 h post-injection, mice were euthanized, and tumors were collected to stain for HIF-1α positivity. Custom MATLAB code was used to assess the HIF-1α positivity of these analyzed tumor sections.

### *In Vivo* Antitumor Study

When orthotopic CT26 tumors were up to 3 mm in diameter, as measured via endoscopy, tumor-bearing mice were randomly assigned to four groups (*n* = 5/group): (1) PBS + interventional white laser (L); (2) RMD + L; (3) PMD + external white laser irradiation (EI); and (4) PMD + L. In groups 2–4, a DCPy dose of 5 mg/kg was utilized. At 6 h post-injection of the appropriate particle solutions, PDT (0.1 W/cm^2^, 20 min) was conducted. The indicated treatments were performed every 4 days for 14 days, with murine body weight being monitored every other day. After this period, mice were euthanized and both tumors and primary organs (kidneys, lungs, heart, liver, spleen) were collected, rinsed in PBS, and fixed with paraformaldehyde for histological analyses. Tumors were weighed, imaged, fixed with 4% neutral-buffered formalin, paraffinized, and cut into 4 μm sections that were then subjected to TUNEL staining and hematoxylin and eosin (H&E) staining followed by imaging with a light microscope (BX51, Olympus, Japan).

### Statistical Analysis

GraphPad Prism 5.0 was used to analyze data, which were compared between groups via Student’s *t*-test. *P < 0.05, **P < 0.01, ***P < 0.001.

## Supporting Information Available

NMR and mass spectra for DCPy, characterization of nanoparticles, stability evaluation of PMD in PBS and FBS, expression of key protein P-selectin on PV and PMD, uptake of nanoparticles by RAW 264.7 cells, *in vitro* cytotoxicity of PMD, hemolysis ratio of PMD at different DCPy concentrations, quantification of H_2_O_2_ in CT26 cells, quantification of HIF-1α immunofluorescent staining, and histopathologic examination (PDF)
