# Supplementary material for: AIEgen-Based Bionic Nanozymes for the Interventional Photodynamic Therapy-Based Treatment of Orthotopic Colon Cancer
Source: ACS Appl Mater Interfaces. 2022 May 11;14(23):26394–403. doi: 10.1021/acsami.2c04210 (PMC9204689; doi:10.1021/acsami.2c04210)
Supplement: Supplementary file 1 — am2c04210_si_001.pdf [file am2c04210_si_001.pdf]

Supporting information

AIEgen-Based Bionic Nanozymes for the Interventional Photodynamic Therapy-Based Treatment of Orthotopic Colon Cancer

Yanhong Duo,[1,6,7,#,*] Meng Suo,[5,#] Daoming Zhu,[5] Zihuang Li,[1,*] Zheng Zheng,[3,4,*] and Ben Zhong Tang[2,*]

[1]Department of Radiation Oncology, the Second Clinical Medical College of Jinan University, 1st Affiliated Hospital of Southern University of Science and Technology, Shenzhen People's Hospital, Shenzhen 518020, China.

E-mail: duoyh@tsinghua-sz.org.

[2]School of Science and Engineering, Shenzhen Institute of Aggregate Science and Technology, The Chinese University of Hong Kong, Shenzhen, Guangdong 518172, China.

E-mail: tangbenz@cuhk.edu.cn.

[3]School of Chemistry and Chemical Engineering, Hefei University of Technology, Hefei 230009, China.

E-mail: zzheng@hfut.edu.cn.

[4]AnHui Province Key Laboratory of Chemistry for Inorganic/Organic Hybrid Functionalized Materials, Anhui University, Hefei 230601, P.R.China.

E-mail:zzheng@hfut.edu.cn.

[5]Department of Electronic Science and Technology, School of Physics and Technology, Wuhan University, Wuhan 430072, China.

[6]Department of Microbiology, Tumor and Cell Biology (MTC), Karolinska Institutet, Stockholm 17177, Sweden.

E-mail:duoyh@tsinghua-sz.org.

[7]Department of Sports Medicine and Rehabilitation, Shenzhen Hospital Peking University, Shenzhen 518036, China.

E-mail: duoyh@tsinghua-sz.org.

[#]Authors contributed equally.

[*]Corresponding authors.

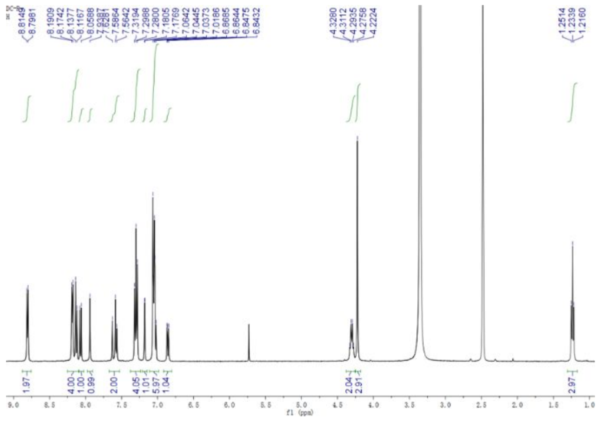

Figure S1. ^1H NMR spectrum of DCPy were recorded on a Bruker ARX 400 NMR spectrometer using DMSO-d_6 as solvent and tetramethylsilane (TMS; $\delta = 0$ ppm) was chosen as internal reference.[1]

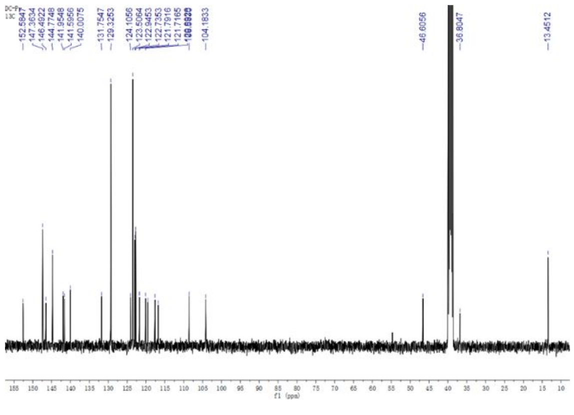

Figure S2. ^{13}C NMR spectrum of DCPy were recorded on a Bruker ARX 400 NMR spectrometer using DMSO-d_6 as solvent and tetramethylsilane (TMS; δ = 0 ppm) was chosen as internal reference. [1]

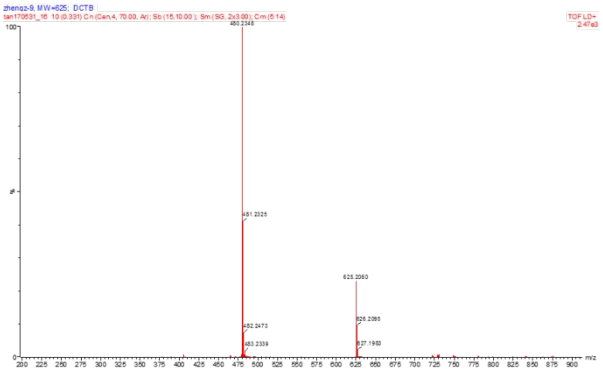

Figure S3. HRMS spectrum of DCPy obtained from a Finnigan MAT TSQ 7000 Mass Spectrometer System operated in a MALDI-TOF mode.[1]

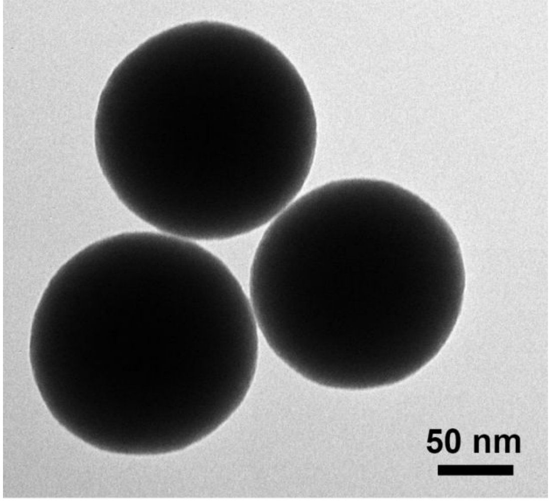

Figure S4. TEM image of $sSiO_2$ nanoparticles.

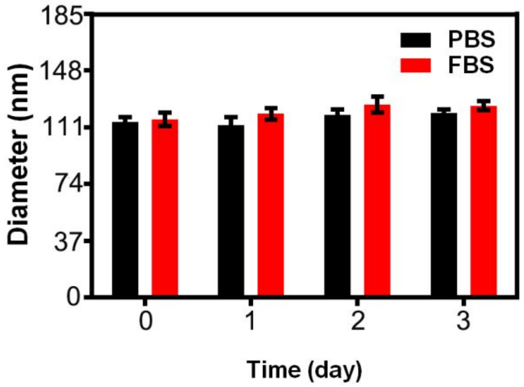

Figure S5. Stability evaluation of PMD nanoparticles in PBS or FBS for 3 days.

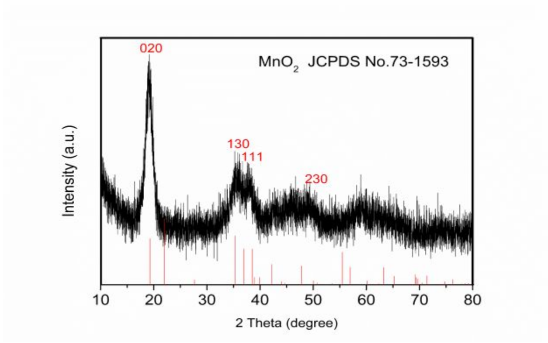

Figure S6. XRD spectrum of MnO_2 coated silica nanoparticle.

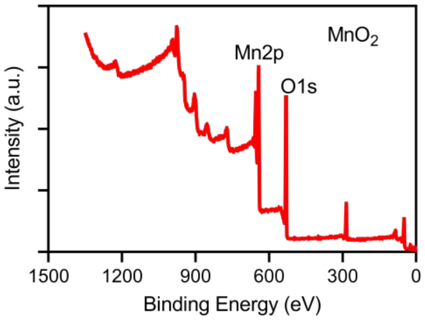

Figure S7. XPS sprectrum of MnO_2 coated silica nanoparticle.

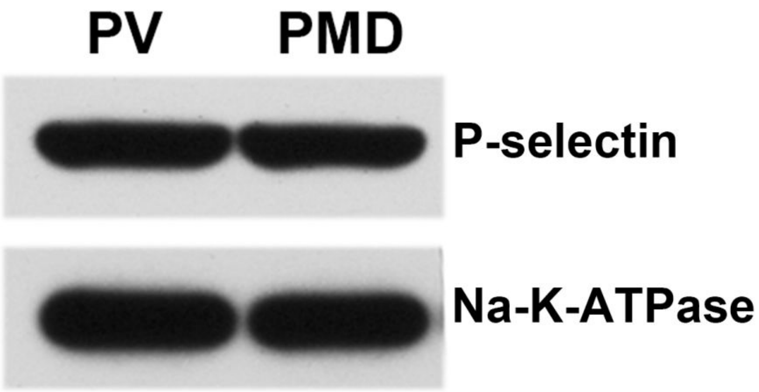

Figure S8. The expressions of key protein P-selectin on PV and PMD.

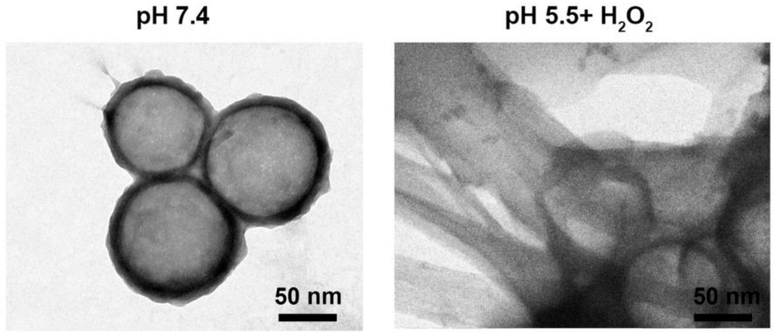

Figure S9. TEM image of PMD after incubation under different conditions.

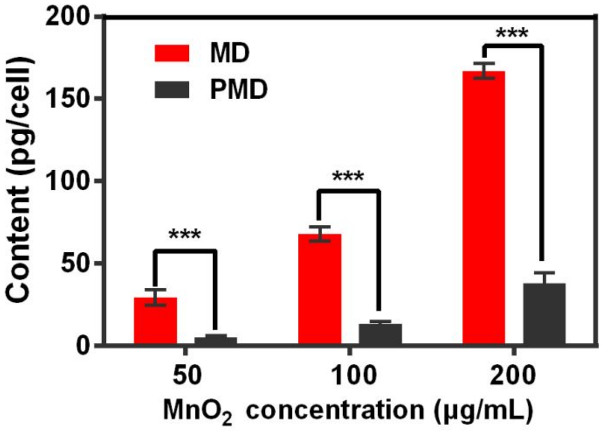

Figure S10. Nanoparticles uptake by RAW 264.7 cells at different concentration.

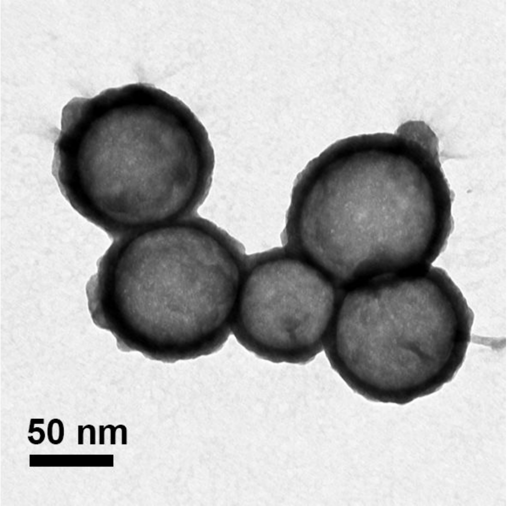

Figure S11. TEM image of RMD.

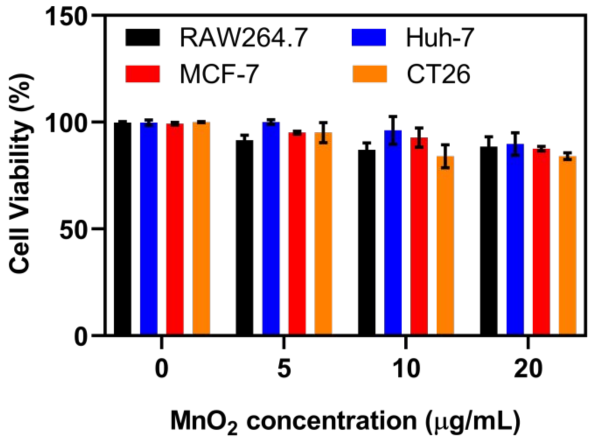

Figure S12. *In vitro* cytotoxicity of PMD on different cells in the absence of irradiation. Data were presented as mean ± SD (n = 3).

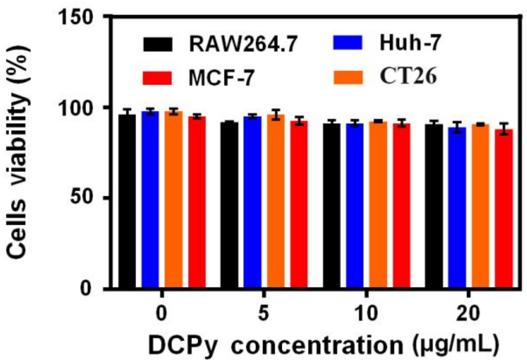

Figure S13. *In vitro* cytotoxicity of PMD on different cells in the absence of irradiation. Data were presented as mean ± SD (n = 3).

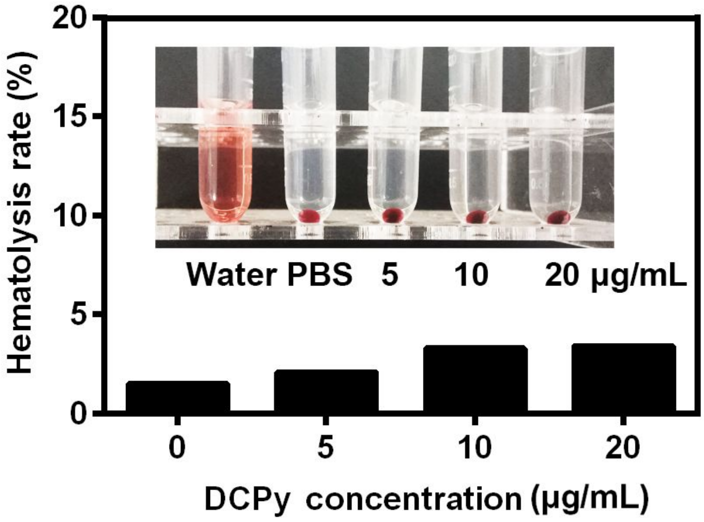

Figure S14. Hemolysis ratio of PMD at different DCPy concentrations. The inset shows the corresponding hemolysis images.

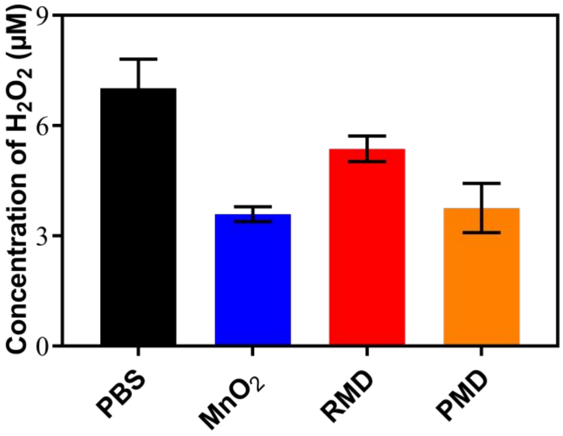

Figure S15. Quantification of H_2O_2 in CT26 cells.

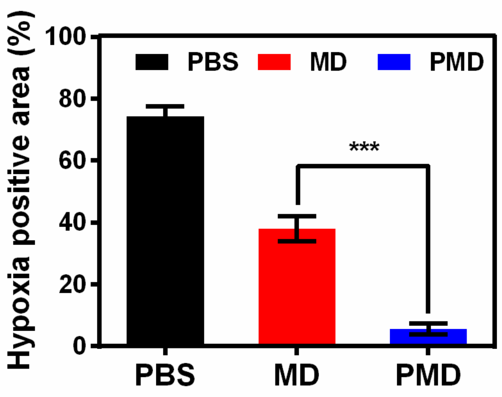

Figure S16. Quantification of HIF-1α immunofluorescent staining. (Student's t test was used, **P < 0.01, ***p<0.001.)

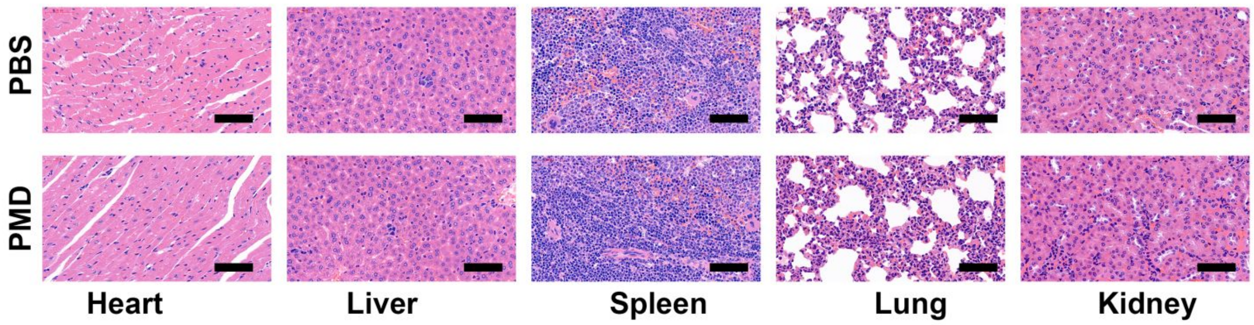

Figure S17. Histopathologic examination of the tissues including heart, liver, spleen, lung, and kidney from BALB/c mice after different treatment. Scale bars = 100 μm.
